# Visual Acuity in Late Adolescence and Future Psychosis Risk in a Cohort of 1 Million Men

**DOI:** 10.1093/schbul/sby084

**Published:** 2018-06-12

**Authors:** Joseph F Hayes, Suzanne Picot, David P J Osborn, Glyn Lewis, Christina Dalman, Andreas Lundin

**Affiliations:** 1 Division of Psychiatry, University College London, London, UK; 2 Department of Public Health Sciences, Unit of Public Health Epidemiology, Karolinska Institute, Stockholm, Sweden

**Keywords:** eyesight, vision, schizophrenia, sibling-design

## Abstract

**Background:**

We aimed to determine whether late adolescent visual impairment is associated with later psychosis.

**Methods:**

We conducted a longitudinal cohort study of Swedish male military conscripts aged 18 or 19 years from January 1, 1974, through December 31, 1997 (*N* = 1140710). At conscription, uncorrected and optometry-lens-corrected distance visual acuity was measured. Participants were then followed up to see if they received an inpatient diagnosis of non-affective psychotic disorder, including schizophrenia (*N* = 10769). Multivariable Cox modeling was used to estimate differences between groups.

**Results:**

After adjustment for confounders, those with severe impairment before optical correction in their best eye (decimal fraction <0.3) had an increased psychosis rate compared to those with normal uncorrected vision (decimal fraction 1.0) (hazard ratio [HR] 1.26, 95% CI 1.16–1.37). Larger interocular visual acuity difference was associated with an increased psychosis rate (adjusted HR 1.49, 95% CI 1.37–1.63 in those with differences >0.5 compared to those with no between eye acuity difference). Individuals with impaired vision that could not be corrected to normal with lenses had highest rates of psychosis (best eye adjusted HR 1.56; 95% CI 1.33–1.82), those with imperfect, but correctable vision also had elevated rates (best eye adjusted HR 1.21; 95% CI 1.15–1.28). Individuals with visual impairment had higher rates of psychosis than their full siblings with normal vision (adjusted HR 1.20, 95% CI 1.07–1.35).

**Conclusions:**

Impaired visual acuity is associated with non-affective psychosis. Visual impairment as a phenotype in psychosis requires further consideration.

## Introduction

Visual deficits have been identified at all clinical stages of psychotic illness in increasing severity, from high risk,^1,2^ and prodromal states,^3^ through first episode,^4^ to chronic schizophrenia.^5,6^ As such, it has been suggested that visual defects represent a phenotype which could contribute to more reliable diagnostic criteria for schizophrenia.^7^

One important measure of visual function is acuity. Visual acuity is a composite measure of the capacity for the eye to accurately focus light on the retina, the integrity of the retina, and the brains ability to interpret the information provided.^8^ While neurological abnormalities have long been a focus of interest in the aetiology of psychosis, it is only more recently via techniques such as electroretinography and optical coherence tomography, that cross-sectional associations between retinal anomalies and schizophrenia have been identified.^6^ Myopia is the most common cause of reduced distance acuity and affects over 20% of the world’s population.^9^ Despite the high prevalence of abnormal acuity and the apparent increase in visual deficits as psychotic illness progresses,^4,5,10^ very little research has examined the potential relationship between visual acuity and psychosis longitudinally, or on a population-level. One study in offspring of mothers with psychosis found that visual dysfunction aged 4 was associated with schizophrenia-spectrum disorders at 22.^1^ In another cohort with family history of psychosis, visual dysfunction before age 13 was associated with psychosis aged 33.^2^ Both of these studies were small and did not account for any potential confounders. The largest study of which we are aware, found a negative association between corrected refractive errors detected during conscription interviews for the Israeli army and future risk of schizophrenia.^11^ This result is at odds with the rest of the literature.^7^

The limited evidence-base is surprising given the large number of studies examining the relationship between hearing loss and psychosis, in which an association is consistently found.^12^ Similarly, a number of theoretical models for psychosis suggest that impaired vision could be a risk factor. Computational models suggest that psychosis can be understood in terms of defective Bayesian processes; ie, abnormal interactions between perceptual inputs and prior beliefs which lead to experiences of false concepts (delusions) or percepts (hallucinations).^13,14^ Other models, such as the social defeat hypothesis,^15^ impaired theory of mind,^16^ and source monitoring errors^17^ are equally applicable to hearing and visual deficits. In particular, the “protection against schizophrenia” model hypotheses that there will be an inverted U relationship between visual impairment and psychosis risk,^7^ as normal vision and absent vision are associated with decreased psychosis risk.^18^ However, to our knowledge, this has not been investigated in a general population cohort.

We used data from Swedish conscript Snellen eye chart tests to examine whether distance visual acuity is associated with future risk of psychotic illness.

## Methods

### Study Design and Setting

Data were extracted from linked national registers, which include data on all people officially resident in Sweden after January 1, 1932, anonymized by Statistics Sweden for research purposes. Eyesight data were derived from the Military Service Conscript Register and the Military Archives of Sweden. Assessments were conducted with nearly all male members of the Swedish population from January 1, 1974, through December 31, 1997, when aged 18 or 19 years. Individuals could only be exempted from assessment by their General Practitioner because of severe disability. This register also includes IQ test results, reviews of physical and mental health, and sociodemographic data. The diagnosis of psychotic illness was extracted from the National Patient Register for all inpatient treatment episodes in Sweden from January 1, 1974, through December 31, 2011. We also extracted data on the total number of psychiatric admissions for each person diagnosed with psychosis, to act as a proxy for illness severity. The Migration and Cause of Death registers were used to define when people left the cohort. All individuals were censored at the earliest of psychotic illness diagnosis date, migration, death date or December 31, 2011. Individuals who had admissions for serious mental illness (SMI) including bipolar disorder, schizoaffective disorder, schizophrenia or other psychotic illness, before their conscription interview were excluded from the analysis, as were individuals who were assessed as having psychosis at the conscription interview. Parents of conscripts were identified in the Multi-Generational Register and linked to the National Patient Register to identify parental SMI. Parental socioeconomic status (SES) data were derived from census information from 1970 through 1990, and linked through the Multi-Generational Register. Full siblings were also identified through the Multi-Generational Register and their exposure, outcome and covariate information was extracted from the other registers. The study was approved by the Regional Ethical Review Board, Stockholm, Sweden, which waived the need for informed consent for these publicly available data.

### Distance Visual Acuity

Uncorrected and corrected visual acuity assessment was completed by an optometrist using the Snellen eye chart placed at 6 meters. The upper testing limit was 1.0. Corrected acuity was assessed using lenses offered by the optometrist (ie, optimal visual acuity). Results were recorded as decimal fractions. In line with previous research, and World Health Organisation guidance,^19,20^ we categorized uncorrected acuity as “normal” (1.0), “mild visual impairment” (<1.0 to >0.5), “moderately impaired” (≤0.5 to ≥0.3), and “severely impaired” (scores <0.3). We categorized optimal visual acuity as “impaired following correction” (scores <1.0) and “normal following correction” (1.0). We assessed the association with psychosis in both eyes separately. Binocular visual acuity is considered to be approximately equal to the better-seeing eye.^21^ We also considered uncorrected visual acuity as a continuous measure. In addition, we categorized interocular acuity difference as no difference, >0.0 to 0.2, >0.2 to 0.5, and >0.5 decimal fractions. Registered blind individuals were exempted from conscription.

### Non-affective Psychotic Illness

Schizophrenia or other non-affective nonorganic psychoses diagnoses were based on Swedish versions of the International Classification of Diseases (ICD-8 to ICD-10). Schizophrenia ICD-8: 295.0-.4, .6, .8, .9; ICD-9: 295.A-.G, .W, .X; ICD-10: F20.0-.3, .5, .6, .8, .9. Other non-affective psychoses ICD-8: 297.0, .1, .9; 298.1-.3, .9; 299.9; ICD-9: 297.B-.D, .W, .X; 298.B-.E, .W, .X; ICD-10: F21; F22; F23; F24, F28, F29. Diagnosis date was defined as the date of the first psychiatric inpatient record. Schizophrenia and non-schizophrenia psychosis diagnoses were also examine separately as outcomes. To account for the varying follow-up time we defined psychiatric admission frequency as the number of admissions per 5 years of follow-up (categorized as <1.0, 1.0 to <2.0, 2.0 to <5.0, ≥5.0).

### Other Covariates

Potential confounding factors were included, based on previous research. Covariates were: age, year of conscription interview, SES (defined by highest parental employment using the Swedish Occupational Class schema and categorized as unemployed, worker, white-collar-workers or business owners),^22,23^ IQ (categorized as <74, 74–81, 82–89, 90–95, 96–104, 105–110, 111–118, 119–126, >126),^24,25^ history of common mental disorder (CMD), including depression or anxiety disorders (from conscription assessment and medical records),^26,27^ parental history SMI history,^28^ alcohol use disorder and substance use disorder (from conscription assessment).^29,30^

### Statistical Analysis

Cox proportional hazards regression analyses were conducted comparing the relative hazards of developing psychotic illness in different categories of uncorrected and corrected visual impairment. We adjusted for potential confounders described above. Analysis of Schoenfeld residuals was completed to test the proportional hazards assumption.^31^ We tested for acuity-IQ and acuity-parental SMI interactions using likelihood ratio tests. We repeated the uncorrected acuity analysis using acuity as a continuous variable and fitted a fractional polynomial model. We ran all models after removing participants who developed psychosis within 5 years of their conscription interview, to assess whether any association was likely to be the result of prodromal psychosis. We carried out similar analyses with the exposure defined as the interocular acuity difference. We conducted a multivariable ordinal logistic regression to assess the association between acuity and frequency of psychiatric hospitalization.

We identified full sibling dyads in the cohort, selecting the nearest-born sibling if there were multiple. We tested whether impaired acuity in the sibling was associated with psychosis in the index participant. This analysis should test for shared genetic liability or common environmental influences for impaired acuity and psychosis. We then identified dyads discordant for normal vs impaired acuity in their best eye. We carried out a multivariable Cox regression adjusting for confounders that could differ between siblings (age, year of conscription interview, birth order, IQ, CMD, alcohol, and other substance use disorders) and accounting for the effect of family using a clustered sandwich estimator. This analysis should partially account for unmeasured genetic, social and lifestyle confounders that are shared within families, as full siblings share up to half their genetic makeup and generally share their early environment.^32^ All analyses were conducted with Stata software (Version 14; StataCorp).^33^

## Results

Of the men attending conscription interviews (*N* = 1229862), 1140710 had interpretable visual acuity tests. Normal acuity (1.0) in the best eye was found in 903227 men (79.18%). Individuals with severe visual impairment were most likely to have parents who were white-collar workers, more likely to have a higher IQ and less likely to have a history of alcohol or substance use disorder (table 1). The mean follow-up for individuals in the cohort was 24.75 years (SD 8.32) There were 10769 new cases of psychosis (including 5522 with schizophrenia) within the follow-up time of 28.46 million person-years (3.78 per 10000 person-years at risk [PYAR]; 95% CI 3.71–3.86). The mean age of first diagnosis of psychotic illness was 29.53 (SD 8.25 y).

**Table 1.** Baseline Characteristics by Uncorrected Visual Acuity in Best Eye

	Normal Acuity (1.0)	Mild Impairment (<1.0 to >0.5)	Moderate Impairment (≤0.5 to ≥0.3)	Severe Impairment (<0.3)
Total, *N* (%)	903227 (79.18)	84663 (7.42)	62678 (5.49)	90142 (7.90)
Age, mean (SD)	18.30 (0.79)	18.32 (0.85)	18.32 (0.79)	18.30 (0.68)
SES, *N* (%)				
Unemployed	25950 (2.87)	2746 (3.24)	1864 (2.97)	2467 (2.74)
Worker	307377 (34.03)	27495 (32.48)	19131 (30.52)	25379 (28.15)
White-collar	414902 (45.94)	40930 (48.34)	32070 (51.17)	49259 (54.65)
Business owner	122880 (13.60)	10167 (12.01)	7280 (11.61)	10005 (11.10)
Missing	32118 (3.56)	3325 (3.93)	2333 (3.72)	3032 (3.36)
IQ group, median	96–104	96–104	96–104	105–110
CMD, *N* (%)	17202 (1.90)	1608 (1.90)	1025 (1.64)	1093 (1.21)
Parental SMI, *N* (%)	31280 (3.46)	3030 (3.58)	2252 (3.59)	2997 (3.32)
Alcohol misuse *N* (%)	1424 (0.16)	112 (0.13)	49 (0.08)	33 (0.04)
Substance misuse *N* (%)	2068 (0.23)	142 (0.17)	72 (0.11)	49 (0.05)

*Note*: SES, socioeconomic status; CMD, common mental disorder; SMI, serious mental illness.

### Uncorrected Vision

**Fig. 1.**
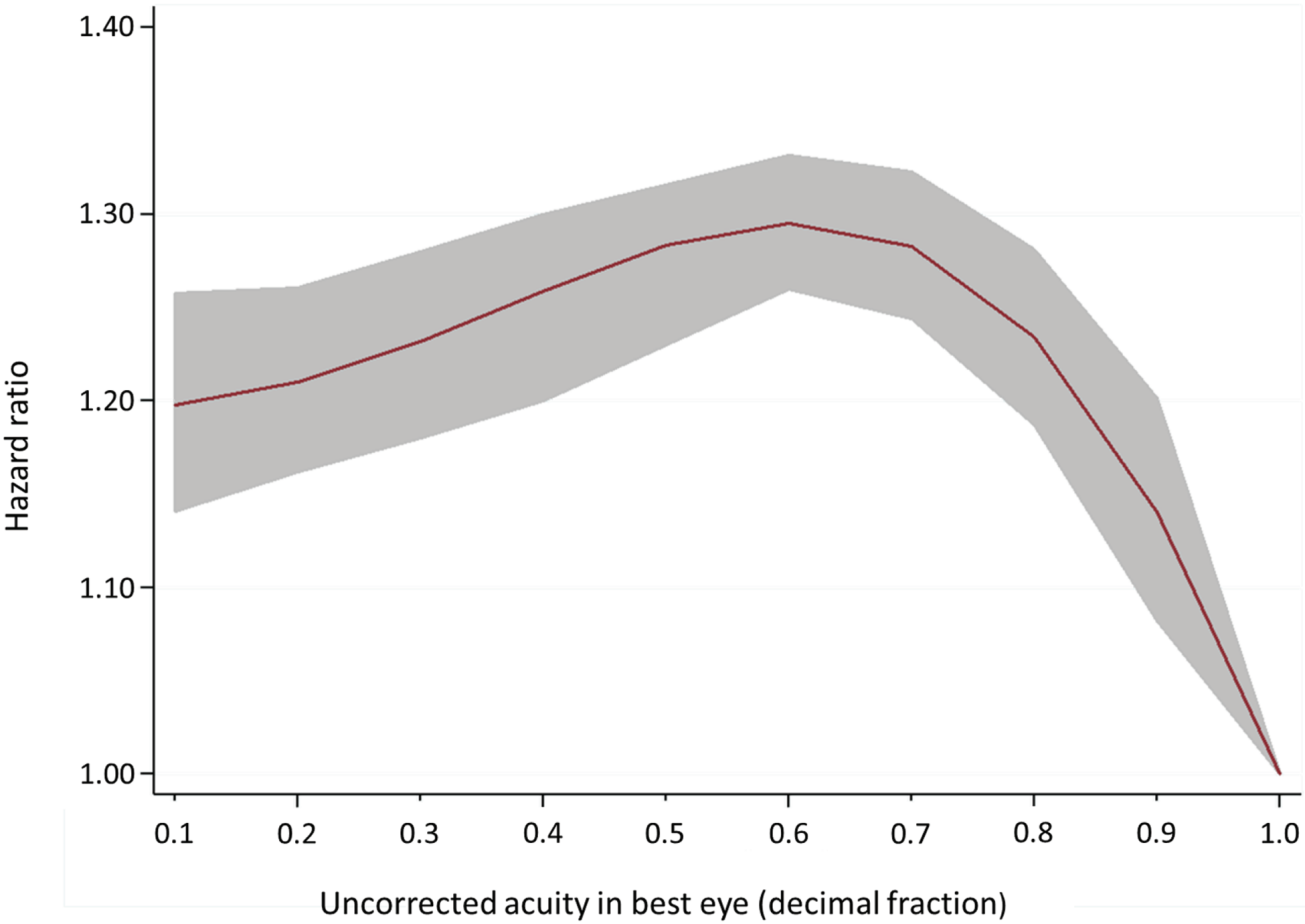
Uncorrected visual acuity and psychosis diagnosis from fully adjusted fractional polynomial model. Hazard ratio and 95% CI.

Impaired acuity in either eye was associated with increased psychotic illness rates (table 2). When using acuity as a continuous measure, for each decimal reduction in acuity there was an increase in rate of psychosis until 0.6 (figure 1). There was no evidence of interaction between acuity and IQ (*P* = .88) or acuity and parental SMI (*P* = .76). Removing individuals who developed psychosis within 5 years of their conscription interview (*N* = 2511) had little effect on the association (best eye fully adjusted hazard ratio [HR] 1.19, 95% CI 1.09–1.28; HR 1.21, 95% CI 1.10–1.34; HR 1.25, 95% CI 1.15–1.37 for mild, moderate, and severe impairment, respectively compared to normal vision). Impaired acuity (<1.0) was associated with both increased rates of schizophrenia diagnoses (adjusted HR 1.31, 95% CI 1.22–1.41) and of other psychoses (adjusted HR 1.17, 95% CI 1.08–1.26). Increasing interocular acuity difference was associated with an increased psychosis rate (table 3). Impaired acuity in the best eye was associated with an odds ratio of 1.13, 95% CI 1.03–1.24 per group increase in 5-year admission rate, after adjustment, with groups defined as <1.0 (*N* = 4906), 1.0 to <2.0 (*N* = 2258), 2.0 to <5.0 (*N* = 2375), ≥5.0 (*N* = 1230) admissions per 5 years. There was no evidence this model violated the parallel regression assumption.

**Table 2.** Association Between Visual Acuity and Non-affective Psychosis

	Best eye				Worst Eye			
	*N* (%)	Events/PYAR	HR (95% CI)		*N* (%)	Events/PYAR	HR (95% CI)	
			Unadjusted	Adjusted^a^			Unadjusted	Adjusted^a^
Uncorrected acuity								
Normal (1.0)	903227 (79.18)	8441/22.97 × 10^6^	1 (Reference)	1 (Reference)	795250 (69.72)	7138/20.14 × 10^6^	1 (Reference)	1 (Reference)
Mild impairment (<1.0 to >0.5)	84663 (7.42)	946/20.27 × 10^5^	1.24 (1.16–1.33)	1.25 (1.16–1.34)	107476 (9.42)	1190/25.97 × 10^5^	1.27 (1.20–1.35)	1.22 (1.14–1.31)
Moderate impairment (≤0.5 to ≥0.3)	62678 (5.49)	609/15.17 × 10^5^	1.07 (0.99–1.17)	1.19 (1.09–1.31)	86659 (7.60)	952/21.42 × 10^5^	1.24 (1.16–1.33)	1.34 (1.24–1.44)
Severe impairment (<0.3)	90142 (7.90)	773/19.48 × 10^5^	1.02 (0.94–1.09)	1.26 (1.16–1.37)	151325 (13.27)	1489/35.74 × 10^5^	1.15 (1.09–1.21)	1.38 (1.30–1.47)
Corrected acuity								
Normal uncorrected (1.0)	903227 (79.18)	8441/22.97 × 10^6^	1 (Reference)	1 (Reference)	795250 (69.72)	7138/20.14 × 10^6^	1 (Reference)	1 (Reference)
Corrected to normal (1.0)	225896 (19.80)	2123/52.12 × 10^5^	1.06 (1.02–1.12)	1.21 (1.15–1.28)	290790 (25.49)	2869/69.38 × 10^5^	1.14 (1.09–1.19)	1.30 (1.24–1.36)
Imperfectly corrected (<1.0)	11511 (1.01)	205/27.75 × 10^4^	1.98 (1.72–2.27)	1.56 (1.33–1.82)	54670 (4.79)	762/13.74 × 10^5^	1.56 (1.44–1.68)	1.38 (1.28–1.50)

*Note*: PYAR, person-years at risk; HR, hazard ratio.

^a^Adjusted for age at conscription, calendar year, IQ, socioeconomic status, common mental disorder, parental serious mental illness, alcohol use disorder and substance use disorder.

**Table 3.** Rate of Psychosis by Interocular Difference in Acuity

	*N* (%)	Events/PYAR	HR (95% CI)	Adjusted^a^
			Unadjusted	
Interocular difference (decimal fractions)				
0	922448 (80.87)	8320/22.96 × 10^6^	1 (Reference)	1 (Reference)
>0.0 to 0.2	114946 (10.08)	1186/27.37 × 10^5^	1.18 (1.11–1.25)	1.19 (1.12–1.28)
>0.2 to 0.5	56059 (4.91)	638/13.80 × 10^5^	1.27 (1.17–1.38)	1.30 (1.19–1.41)
>0.5	47257 (4.14)	625/13.18 × 10^5^	1.37 (1.26–1.48)	1.49 (1.37–1.63)

*Note*: PYAR; person-years at risk, HR; hazard ratio.

^a^Adjusted for age at conscription, calendar year, IQ, socioeconomic status, common mental disorder, parental serious mental illness, alcohol use disorder, and substance use disorder.

We conducted additional analyses in which we compared full sibling dyads (table 4). Sibling acuity was not associated with index participant psychosis (adjusted HR 1.00, 95% CI 0.91–1.10). In discordant sibling-pairs there were elevated psychosis rates in siblings with impaired visual acuity (adjusted HR 1.23, 95% CI 1.07–1.43).

**Table 4.** Sibling Dyad Analyses

	*N* (%)	Events/PYAR	HR (95% CI)	Adjusted^a^
			Unadjusted	
Acuity of sibling of index participant				
1.0	289683 (79.80)	2536/72.86 × 10^5^	1 (Reference)	1 (Reference)
<1.0	73341 (20.20)	606/17.34 × 10^5^	0.98 (0.90–1.07)	1.00 (0.91–1.10)
Discordant sibling pairs				
1.0	49045 (50.00)	393/11.95 × 10^5^	1 (Reference)	1 (Reference)
<1.0	49045 (50.00)	446/11.63 × 10^5^	1.16 (1.01–1.32)	1.23 (1.07–1.43)

*Note*: PYAR; person-years at risk, HR; hazard ratio.

^a^Sibling acuity analysis adjusted for index participant acuity age at conscription, calendar year, IQ, common mental disorder, alcohol use disorder and substance use disorder, discordant sibling pair analysis adjusted for age at conscription, calendar year, IQ, common mental disorder, alcohol use disorder and substance use disorder. All models account for the effect of family using a clustered sandwich estimator.

### Corrected Vision

There were increased psychosis rates in individuals who had impaired eyesight corrected to normal (best eye adjusted HR 1.21, 95% CI 1.15–1.28), and higher rates in those who had their eyesight imperfectly corrected (best eye adjusted HR 1.56, 95% CI 1.33–1.82) compared to those with normal vision without correction (table 2). Removal of individuals potentially in the prodromal phase of psychotic illness (ie, psychosis within 5 y) had minimal effect (corrected to normal best eye adjusted HR 1.23, 95% CI 1.18–1.30; corrected to imperfect best eye adjusted HR 1.49, 95% CI 1.33–1.67).

## Discussion

As far as we are aware, this is the largest study of the association between visual acuity in late adolescence and future risk of psychotic illness. We studied over 1 million men with up to 38 years of follow-up. We found a gradient in the relationship between uncorrected distance acuity and psychotic illness, which was not explained by age, calendar period, SES, IQ, CMD, parental SMI, alcohol or substance misuse. Correction of acuity to normal did not reduce the risk of psychosis to baseline. Exclusion of individuals developing psychosis within 5 years of their conscription interview, to remove potential influence of prodromal psychotic symptoms on test performance, did not affect our results. Increase in the interocular difference in acuity was associated with psychosis. Impaired acuity was also associated with increases in hospitalization frequency in the group with psychosis, suggesting illness severity may additionally be linked to acuity. We found a similar association in siblings discordant for visual acuity impairment, reducing the possibility that the observed association is due to unmeasured or residual confounding. Sibling acuity was not associated with index participant psychosis, again suggesting that the association is not through a shared genetic liability or shared environment. Our results support the “protection against schizophrenia” model developed by Landgraf and Osterheider.^7^

Despite studies of psychotic symptoms in old age,^34^ and cross-sectional studies of visual impairment in patients with psychosis,^4,5,10^ there is relatively little previous literature clarifying the temporal relationship between acuity and psychosis. The study most similar to ours, which used an Israeli military service cohort, found that a binary definition of corrected refractive error at aged 17 was related to reduced, rather than increased, risk of schizophrenia.^11^ This study examined schizophrenia specifically, and found a very low prevalence of refractive errors (5% in the “control” group and 0.9% in the schizophrenia group). This is at odds with a study using the same Israeli conscription data, which reports approximately 20% myopia.^35^ Rates of schizophrenia diagnosis are not presented, and the paper appears to use a combination of case-control and cohort approaches, making comparison with our study challenging. The other cohort studies of which we are aware, were small in comparison, but found a positive association between visual disturbance earlier in childhood and adult psychosis.^1,2^

That the association between impaired acuity and psychosis/schizophrenia may be causal accords with many models for psychosis.^13–17^ However, our data do not permit the nature of this association to be specified or whether a third, as yet unknown variable, might be related to the association. People with impaired acuity have impairments in reading, face recognition, stereoacuity, and performance of everyday vision-related tasks.^36^ Possible routes for the impact of impaired acuity are through impaired schema development and impaired social cognition. It has been hypothesized that visual perceptual anomalies can interrupt continuity in the experience of self, in particular theory of mind and aspects of self-monitoring, leading to psychosis.^37^ This is supported by research in experimental psychopathology and cognitive neuroscience.^38^ Social perception has been found to mediate the relationship between visual processing and functional status in schizophrenia.^39^ One argument against the causal effect of visual impairment is that individuals with a correctable impairment remain at increased risk. However, we do not know how long individuals lived with uncorrected visual impairment before detection and correction.

Alternately, there may be common causes that poor acuity and psychosis share. Schubert et al., measured visual disturbance in 4-year olds, and found that all children with visual dysfunction had neurological abnormalities aged 6, and suggested that their results supported a neurodevelopmental aetiology hypothesis.^1^ In our study, it seems unlikely that impaired visual acuity at 18 reflects quantifiable neurological abnormalities in 20% of the population. The majority of impaired acuity at this age is likely to be because of myopia.^40^ Therefore, there may be particular vulnerability to psychosis in young children with early visual impairment, related to neurodevelopmental abnormalities, but potentially other pathways in later life related to myopia. For example, experimental and epidemiological evidence suggests that myopia risk is inversely associated with outdoor light intensity and time spend outdoors.^40^ It has been postulated that increased light might protect against myopia because of increased transmitter dopamine^41^ or increased vitamin D.^42^ Schizophrenia and other non-affective psychosis has long recognized to be associated with increased latitude and reduced sunlight exposure.^43^ Similarly, vitamin D has been implicated as playing a role.^43^ That dopamine dysfunction is the final pathway to psychosis is one of the most enduring hypotheses in psychiatry.^44^ The argument against myopia and psychosis sharing a common cause such as this is that the association remained when we adjusted for a number of confounders, and when we compared siblings discordant for impaired acuity; these siblings are likely to have experienced similar environmental exposures. It is notable that countries that countries with increasing incidence of myopia^45^ also report increasing psychosis rates.^46^ Scandinavian rates of myopia and psychosis are likely have been stable over our study period.^47,48^

### Strengths and Limitations

Beyond the size and long follow-up of this cohort, its strength is it represents nearly all men in Sweden. Conscription was mandatory until 2000, and only 3%–5% were exempt from assessment due to severe disability (including blindness).^49^ The prevalence of impaired visual acuity (20%) and the incidence of psychosis (4 per 10000 PYAR) reflect rates in other European countries,^9,50,51^ suggesting our findings may generalize to other populations. The relatively young age at which acuity was assessed reduces the risk of reverse causation. In addition, we were able to control for a number of potential confounding factors.

Our study has a number of limitations. A one-off measure using a Snellen chart is a potentially imperfect reflection of visual acuity. It is a subjective test, reliant on the individual’s engagement with the task, so there is potential for fabrication of results.^52^ However, any fabrication is likely to be non-differential with regards to the psychosis outcome. In addition, the test used at conscription had an upper limit of 1.0, whereas some extend to 2.0, potentially providing further insights into our hypothesis. Conversely, the strength of the Snellen test is that it is simple to administer, used worldwide, and is considered the primary indicator of the magnitude of functional impairment due to vision loss.^53^ The age at which individuals developed acuity problems and how quickly they were detected and corrected is unknown. If there were long periods with uncorrected vision, this could potentially explain why even individuals with vision that could be corrected to normal at 18 remained at increased risk of psychosis. Visual correction via glasses wearing has been associated with bullying,^54^ which in turn is associated with psychosis.^55^

Outcome misclassification because of the use of hospital records is unlikely in schizophrenia, because more than 90% will be admitted to hospital at some point during their illness.^56,57^ However, there is potentially more variability in other psychosis diagnoses. For example, in Stockholm 75% of incident psychosis cases were treated in inpatient care by the end of the follow-up period of this study, before this the percentage was higher due to limited community services.^58^ If this misclassification were non-differential with respect to acuity, our results would represent an underestimate of the true association. Similarly, there may be issues with using hospitalization frequency as a proxy for severity, because of changes in service provision. SES was the only variable with missing data (<5%) and therefore a complete case analysis should produce unbiased results.^59^

It is possible that our results contain residual or unmeasured confounding. Generally, factors which increase the risk of psychosis will be associated with low SES and lower IQ, but this is not the case for visual acuity. Unmeasured confounders that may increase both myopia and psychosis are urbanicity^9,60,61^ and latitude.^62,63^ However, siblings are likely to share both these exposures and our discordant sibling analysis produced similar results suggesting that unmeasured shared genetic and environmental exposures do not explain our findings. To rule out shared genetic architecture between acuity and psychosis would require genome-wide association data on all individuals. In addition, our analysis may still be limited by unmeasured time-varying or sibling-varying covariates within families. Finally, our population only included men and it is not clear if we can extrapolate our findings to women; however, sex generally does not modify the effect of other psychosis risk factors.^64^

## Conclusion

Impaired visual acuity in men aged 18 is a precursor to future risk of psychosis. This does not appear to be related to effects of psychosis prodrome, and is not explained by genetic or familial environmental factors. Given the extensive hunt for phenotypes and biological markers, impaired vision has been relatively unexplored in the aetiology of psychosis. Further exploration of timing and trajectories of visual acuity changes, along with clarification of the association of psychosis with specific subtypes of acuity deficit (ocular vs neurological)^6^ may shed light on the phenomenology and symptomology of psychotic illnesses.

## Funding

This study was supported by grant MR/K021362/1 from the Medical Research Council and grant 523-2010-1052 from the Swedish Research Council. J.F.H., G.L., and D.P.J.O. are supported by the UCLH NIHR Biomedical Research Centre. The funding sources had no role in the design and conduct of the study; collection, management, analysis, and interpretation of the data; preparation, review, or approval of the manuscript; and decision to submit the manuscript for publication.
